# Perspectives on clinical use of bioimpedance in hemodialysis: focus group interviews with renal care professionals

**DOI:** 10.1186/s12882-018-0907-4

**Published:** 2018-05-23

**Authors:** Jenny Stenberg, Catrin Henriksson, Magnus Lindberg, Hans Furuland

**Affiliations:** 1 Department of Medical Sciences, University Hospital, Entrance 40, 751 85 Uppsala, Sweden; 2 Department of Medical Sciences, Uppsala University, Uppsala, Sweden; 3 Department of Health and Caring Sciences, University of Gävle, Gävle, Sweden; 4 Department of Public Health and Caring Sciences, Uppsala University, Uppsala, Sweden

**Keywords:** Barriers, Bioimpedance, Dry weight, Facilitators, Focus groups, Renal dialysis

## Abstract

**Background:**

Inadequate volume control may be a main contributor to poor survival and high mortality in hemodialysis patients. Bioimpedance measurement has the potential to improve fluid management, but several dialysis centers lack an agreed fluid management policy, and the method has not yet been implemented. Our aim was to identify renal care professionals’ perceived barriers and facilitators for use of bioimpedance in clinical practice.

**Methods:**

Qualitative data were collected through four focus group interviews with 24 renal care professionals: dieticians, nephrologists and nurses, recruited voluntarily from a nation-wide selection of hemodialysis centers, having access to a bioimpedance-device. The participants were connected to each other and a moderator via equipment for telemedicine and the sessions were recorded. The interviews were semi-structured, focusing on the participants’ perceptions of use of bioimpedance in clinical practice. Thematic content analysis was performed in consecutive steps, and data were extracted by employing an inductive, interactive, comparative process.

**Results:**

Several barriers and facilitators to the use of bioimpedance in clinical practice were identified, and a multilevel approach to examining barriers and incentives for change was found to be applicable to the ideas and categories that arose from the data. The determinants were categorized on five levels, and the different themes of the levels illustrated with quotations from the focus groups participants.

**Conclusions:**

Determinants for use of bioimpedance were identified on five levels: 1) the innovation itself, 2) the individual professional, 3) the patient, 4) the social context and 5) the organizational context. Barriers were identified in the areas of credibility, awareness, knowledge, self-efficacy, care processes, organizational structures and regulations. Facilitators were identified in the areas of the innovation’s attractiveness, advantages in practice, and collaboration. Motivation, team processes and organizational capacities appeared as both barriers and facilitators.

**Electronic supplementary material:**

The online version of this article (10.1186/s12882-018-0907-4) contains supplementary material, which is available to authorized users.

## Background

Assessing hydration status and achieving an adequate dry weight in dialysis patients is a delicate task; morbidity and mortality, primarily due to cardiovascular disease, remain unacceptably high, and emerging evidence suggests inadequate volume control as a main contributor [1–3]. A golden standard for dry weight assessment is absent, and finding practical and reliable tools to ascertain dry weight is a prioritized research area [4, 5].

Traditionally fluid volume status is based on clinical examination, but a number of technologies are now available to aid assessment [6, 7]. Bioimpedance is a non-invasive method that analyzes the electrical resistance and reactance of human tissue. Certain properties of the tissue can be measured: fluid overload, but also normally hydrated lean tissue mass and normally hydrated adipose tissue mass [8, 9]. In recent randomized controlled trials, regression of left ventricular mass index, decrease in blood pressure, improved arterial stiffness and improved survival were demonstrated in hemodialysis patients when bioimpedance spectroscopy was used to guide dry weight adjustments [10, 11]. However, although guidelines welcome novel technical investigations, and a growing body of studies advocates use of bioimpedance to guide dry weight adjustments, researchers exhort for caution when applying results from any technical tool [4, 12–15]. The hemodialysis population consists of heterogeneous and often fragile patients, and since most studies selection criteria are restrictive; sicker patients such as those with implants, major amputation and those with life expectancy of less than one year are usually excluded, thus there are challenges in the clinical application of bioimpedance [16–20]. Cross-sectional studies show that bioimpedance devices are available in several hemodialysis centers, but only about 25% of the units use it regularly in dry weight assessment, most units still lack established guidelines for clinical use of bioimpedance, hence access to a device is not necessarily associated with improved volume control. [21, 22]. For implementation of innovations to be successful, it is crucial to acquire a good understanding of the problem, the target group, its setting and the obstacles to change [23–25]. There is still very little information in published literature as to how well fluid volume management is done in practice and analysis of reasons for professionals not adopting new innovations. The aim of this study was to identify renal care professionals’ perceived barriers and facilitators for use of bioimpedance in clinical practice.

## Methods

### Study design

The study has a qualitative explorative design. Data were collected through focus group interviews. The third step of the Grol and Wensing Implementation of Change Model, which involves problem analysis of the target group and setting, was used as a theoretical framework [26]. The first two steps of the model involve determination of targets for improvement and assessment of the actual performance in practice, those steps were carried out by a previous study [21].

### Participants and setting

We planned for four focus groups with approximately six to eight participants in each group. Focus groups should offer homogeneity with sufficient variation among participants to allow for contrasting opinions [27], thus individuals from a variety of professional backgrounds that may benefit from aid of bioimpedance in renal care – dieticians, nephrologists and nurses, were invited to participate in the study. Participants were recruited from a purposive selection of 13 hemodialysis units of different sizes; units with access to a bioimpedance device, but reporting different levels of use: frequent (*n* = 6), occasional (*n* = 3) or rare (*n* = 4) [21], located in four Swedish regions, inhabiting 70% of the total population. The number of patients treated per unit range from below 20 to close to 200. Due to unavailability of participants for the interview dates two units dropped out, thus, in the final sample, four university hospital units (UHU), six county hospital units (CHU) and one satellite unit (SU) were represented. The included units employ 30 nephrologists and 240 nurses altogether, and in average one dietician per unit.

Presumptive participants received an invitation by e-mail with a short introduction to the study and the procedure of confidentiality, then the first line managers of the addressed units were asked to identify two volunteering renal nurses, whilst dieticians and nephrologists were recruited via national and regional networks. In total, 25 renal care professionals volunteered and gave informed consent to participate, although one nephrologist was later prevented from attending. As hierarchical structures and different levels of education may hinder participants from expressing their opinions [27, 28], the physicians were gathered in a separate group, but for contrast, each focus group included participants from two to four different hospitals. In total, four sessions, each with four to nine participants, were conducted.

### Data collection

A semi-structured questioning route was developed [27, 29, 30]. To evaluate the questioning route and technical facilities, two pilot interviews were conducted. The definitive main questions were:

- Describe how you use bioimpedance in your everyday practice – is it as you planned for?
- What advantages or barriers have you experienced in practice?
- How could use of bioimpedance be improved in your clinic?

An experienced qualitative researcher conducted the focus group interviews, and an assisting moderator was present in all sessions. The respondents gathered in conference rooms at their local hospitals and were connected to the other focus group participants and the moderator via equipment for telemedicine. They were informed that the moderator has a medical background, but no experience with hemodialysis care. Audio recording was used, and in three sessions visual recording was used too. Each session lasted approximately 30 min, and immediately following the interview sessions the records were transcribed verbatim.

### Analysis

**Fig. 1:**
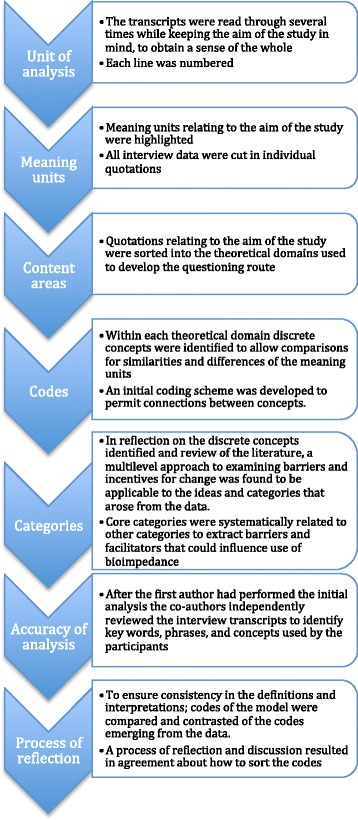
Procedure of content analysis. The analysis process was continual and performed in consecutive steps

The transcripts were read through several times and coded for thematic content analysis in order to inductively derive concepts and core themes from the data. The analysis process was continual and performed in consecutive steps [27, 31, 32] (Fig. 1). Quotations relating to the aim of the study were initially sorted into theoretical domains [30]. Next, within each theoretical domain, discrete concepts were identified to allow comparisons for similarities and differences, and a coding scheme was developed to permit connections between concepts.

In reflection on the discrete concepts identified and after review of the literature, a multilevel approach to examining barriers and incentives for change [23] was found to be applicable to the ideas and categories that arose from the data. The final step involved systematically relating core categories to other categories to extract barriers and facilitators that could influence use of bioimpedance on multiple levels. The first author performed the initial analysis and then the co-authors independently reviewed the interview transcripts to identify key words, phrases, and concepts used by the participants to enhance accuracy of the analysis. The research team discussed the results of the analysis until consensus was reached.

## Results

Determinants for use of bioimpedance in hemodialysis were identified on five levels (Table 1): 1) the innovation itself, 2) the individual professional, 3) the patient, 4) the social context and 5) the organizational context. The different themes of the levels are described and illustrated with quotations from the focus groups. Quotations are identified with the participant’s profession and type of clinic. Table 2 presents descriptive data of the participants.

**Table 1:** Overview of barriers and facilitators on five levels

Level 1–5	Categories:
Type of Determinant	Barriers (−) or Facilitators (+)
1. Innovation	
Credibility	− Measurement is dismissed if it is not supported by user’s perception
	− Difficult to interpret measurement of patients with abnormalities
Attractiveness	+ Feelings of curiosity, satisfaction, excitement
Advantages in practice	+ Has aroused an interest in hydration status in the team, and has given new insights
	+ Software facilitates interpretation of measurement and communication with the patient
2. Individual professional	
Awareness	− The intervention has not been introduced systematically or strategically
	− Continuing education system is insufficient or missing
Knowledge	− Insufficient clarity in recommendation (about limitations and restrictions of utilization) − Lack of pre-existing knowledge or expertise about assessment of hydration status (among nurses)
Motivation	+ Users feel ownership over the initiative and are motivated to develop strategies for use
	− Some are not convinced about the benefits
Self-efficacy	− Concerns about misjudgment – due to lack of skills, experience and decision aids
3. Patient input	
Knowledge	− Patients do not believe in the method
Preferences	− Patients do not want to change routines
Motivation	+ Patients with limited care initiate measurement
4. Social context	
Collaboration	+ Dieticians can contribute knowledge
Team processes	+ Nurses take initiative to measure, then consult the physician to discuss the dry weight
	− Physicians do not trust or follow up results
5. Organizational context	
Capacities	+ In small units the use of bioimpedance has been implemented successfully on oral agreement
	− High workload and shortage of trained staff
Care processes	− The need to wait for the device if someone else is using it interrupts work flow
Structures	− Lack of routine or large variations in routines between units
Regulations	− Isolation of patient with multi-drug resistant infection

**Table 2:** Participant characteristics stratified by profession. Numeric data presented as median and interquartile range

	Dieticians	Nephrologists	Nurses	All participants
N	4	4	16	24
Proportion of represented study population	36%	14%	7%	8%
Men	0%	75% (*n* = 3)	13% (*n* = 2)	21% (*n* = 5)
Age (years)	32 [28,36]	60 [52,63]	44 [37,54]	44 [35,56]
Years in profession	8 [4,11]	31 [25,37]	14 [9,19]	15 [8,26]
Years in current clinic	4 [1,9]	19 [12,25]	10 [3,16]	11 [3,16]

### Innovation

#### Barriers

Several users had been enthusiastic about bioimpedance initially, but expressed declining confidence in the method. If a result was not supported by clinical assessment or other methods for assessment of hydration status, it would be rejected. While individuals considered it a strength to possess a critical mindset, other participants feared assessment of measurement would be arbitrary. Evaluations of bioimpedance measurements on patients who were malnourished, amputees, non-Caucasians, children, body builders, patients with chromosomal abnormalities or with implants were particularly troublesome. Several participants were interested in using bioimpedance to assess nutritional status, but considered validation and reliability insufficient.

##### Credibility

ᅟ

In the beginning we had great faith in it [bioimpedance], and we adjusted the dry weight almost to the hectogram on what it showed, but then reality caught up with us. It turned out it didn’t fit so well [...]. It hasn’t been very useful recently. When a measurement is consistent with our assessment, we think it’s correct, but when it isn’t consistent with our perception, we don’t rely on it. [Nephrologist, CHU]

Some people may have very skinny legs and a corpulent upper body, and then it [bioimpedance] doesn’t work. One must always consider: who is in front of me? Is this really plausible? [Nurse, CHU]

For some, particularly bodybuilders, it’s difficult to get anything out of the bioimpedance measurement. That it doesn’t give any results at all, for example. [Nurse, UHU]

We’ve tried to use it to measure fat mass and fat-free mass, but we’ve found the reliability insufficient, so it hasn’t been of any value. [Dietician, CHU]

#### Facilitators

Use of bioimpedance was associated with feelings of curiosity and excitement; it put dry weight determination on the agenda and provided new insights. Bioimpedance had been particularly helpful in identifying over hydration in cases of young, tall patients with severe hypertension but no visible signs of over hydration. Participants described satisfaction for being able to bring relief to patients by eliminating symptoms of under hydration by increasing dry weight by several kilograms with support of bioimpedance. Although some participants identified insufficient reliability regarding the measurement of nutritional status as a barrier, others found the ability to measure nutritional status an incentive for use, since they considered there were no other objective methods available. Software for visual imaging of changes in body composition over time in a graph was considered a helpful educational tool in interactions with patients.

##### Advantages in practice

ᅟ

You don’t always have to reach absolute dry weight, but you can stay at plus one and a half [liter] and just go on, if the patient is well. So, from that point of view it has of course been useful, and yes it may have given a little more insight into dry weight management, and how to determine, and also the possibility to customize. [Nephrologist, CHU]

Nurses have become much more involved in dry weight. They measure on their own initiative before asking the doctor. Since the machine arrived, discussions regarding dry weight have become much more active. [Nephrologist, CHU]

It also allows us to assess nutritional status and muscle mass, and in some cases it is very important, to see: ‘the patient is keeping weight but losing muscle mass’, and that is a very early warning sign that something is not right. And that is very positive and cannot be done in any other way. [Nephrologist, CHU]

I've actually used it for another purpose too; there’s a patient who's always getting depleted all the time. You cannot have a good discussion with him. But when I do this [bioimpedance measurement], I can show on paper: ‘this muscle mass exists, this fat mass is present, and no water almost.’ Then it may be easier to […] make him understand […] that you cannot lose weight by removing fluids. [Nurse, CHU]

##### Attractiveness

ᅟ

[…] One previous patient, a tall, thin young man, had high blood pressure. We couldn’t understand why […] and he received various drugs, because he had no edema [...]. But then, according to bioimpedance, he had eight liters of fluid overload [...]. When we were gradually able to reduce his weight, he could stop taking many of his blood pressure medications, which is very welcome!. [Nurse, UHU]

### Individual professional

#### Barriers

In several units, bioimpedance had not been introduced systematically or strategically, and the continual education was insufficient, lacking, or dependent on the interest or commitment of certain individuals. Since there was insufficient clarity regarding recommendations, study participants had different perceptions of the limitations and restrictions of use; e.g. some units used bioimpedance in patients with certain pacemakers while others stated that use of bioimpedance was incompatible with all pacemakers. Some nurses and dieticians expressed limited self-efficacy and feared that incorrect performance due to lack of skill and experience in measuring would contribute to misjudgment of hydration status. All professional groups repeatedly emphasized the importance of experience with assessing hydration status, and the urge not to rely on bioimpedance solely. Some nurses acknowledged lack of preexisting knowledge about the distribution of body fluids, especially when affected by malnutrition, inflammation and age, as a barrier for interpretation of the bioimpedance measurement. Some questioned whether bioimpedance could improve dry weight determination at all.

##### Awareness

ᅟ

I attended a lecture about these measurements, but then it had only been performed on non-kidney patients, but so I don’t know if research has been performed in individuals with kidney disease. [Nephrologist, CHU]

We have had someone from [the company], but I don’t know if I got much wiser afterwards, because you want to talk to, you would like to learn from a nurse, who works with patients, with case reports, someone who speaks the same language. [Nurse, UHU]

##### Knowledge

ᅟ

We’ll write it down [ICV / ECV] but then if we can all interpret it or even have a look at it that is something different. [Nurse, UHU]

We’re not allowed to measure on anyone who has a pacemaker, regardless of what kind, just so there won’t be any mistakes. And we don’t measure amputated patients unless it’s absolutely necessary; in those cases, we’ve measured and used this table [for correction] in order to find the right dry weight, but it feels a little less reliable. [Nurse, UHU]

##### Motivation

ᅟ

Of course, you could do it more regularly, but we haven’t really felt the need. [Dietician, CHU]

##### Self-efficacy

ᅟ

Then I think, maybe there has been an uncertainty too, with the device itself that, some may not feel confident, and then maybe they don’t bother to measure. [Nurse, UHU]

[...] You might do a measurement one week, and then the next week the patient may have lost four kilograms, and then the third week it’s completely off the rails. [...] That’s a little hard to know how to deal with. If you should adjust the dry weight, [...] which measurement should you trust?. [Nurse, SU]

#### Facilitators

Study participants felt ownership over the initiative to use bioimpedance and were motivated to develop strategies for use, not least to assess nutritional status objectively, which was considered impossible to do otherwise. Nurses identified bioimpedance as a particularly helpful aid for less experienced colleagues.

##### Motivation

ᅟ

There’s so much more to develop. In addition to determining dry weight, you could use it for nutritional status. And then there are several other groups of patients who would benefit from a measurement. [Nephrologist, CHU]

I’ve noticed that many new [nurses] like to use it because it’s easy. Maybe they haven’t been working for a long time, and thus have less experience using other methods to assess dry weight. [Nurse, UHU]

### Patients’ input

#### Barriers

Participants’ perceptions were that only a minority of patients was reluctant to have their dry weight determined using bioimpedance, because the measurement did not support their own apprehension. A few respondents found that patients were unwilling to postpone start of hemodialysis treatment with approximately 15 min due to the recommendation to rest in a supine position before measurement. Some units did not recognize the delay as a problem since they did not follow the recommendation.

##### Knowledge

ᅟ

Some patients say no. They don’t want to see it; they don’t believe in it. They have their own idea [about dry weight]. [Nurse, CHU]

##### Preferences

ᅟ

Some patients react to the recommendation to rest [before measurement]. They come and want to be connected to dialysis straight away. [Nurse, CHU]

Then you should lie down and rest for 15 to 20 minutes before [...] Try to make a dialysis patient to do that. [Dietician, SU]

#### Facilitators

Patients with limited care and greater involvement in their treatment could initiate a measurement, and use of bioimpedance could contribute to patients’ empowerment.

##### Motivation

ᅟ

In the self-dialysis unit there is often [...] a difference of opinion between patients and physicians regarding ultrafiltration, and then patients themselves might suggest: ‘can’t you do that measurement?’ And often, the patient is actually right. [Nurse, UHU]

My patients may ask, at times, then: What does it [fluid management graph] look like? [Nurse, CHU]

### Social context

#### Barriers

Some nurses expressed frustration because physicians did not trust or follow up results. Use of bioimpedance thus felt meaningless.

##### Team process

ᅟ

Since it’s not followed up, why bother measuring? Some doctors don’t understand it and then they don’t trust the results, and it’s not followed up. So we [the nurses] do measurements, but nothing changes and then it feels meaningless. [Nurse, UHU]

The entire team should be involved, including the physicians, those who... It should not depend on the interest of a few professionals; it's supposed to be the same for all patients, an opportunity to get the best care possible – if you believe this is an improvement. [Nurse, CHU]

#### Facilitators

Dieticians had preexisting knowledge about body composition from their training, and physicians acknowledged dieticians’ contribution in interpreting bioimpedance as highly valuable. Nurses were often prime initiators of measurement, and physicians appreciated it when nurses had performed measurements before discussing dry weight with them. In some units, nurses would adjust dry weight independently with guidance from bioimpedance, while in other units; the physician only performed dry weight determinations.

##### Collaboration

ᅟ

We have a very experienced dietician, who helps us, and she’s tested [...] in patients with prostheses and with various electrodes; we get a lot of support from her. So we know when to expect errors too, and that helps a lot. [Nephrologist, CHU]

##### Team process

ᅟ

In our unit, it is usually the nurses who measure on their own initiative. They are quite independent, determine dry weights and such, they consult us, the physicians only if they feel uncertain. And if they are uncertain, they usually run a BCM and then discuss with us, so it's usually their initiative. [Nephrologist, CHU]

Usually, we the nurses initiate measurements. Confidence among physicians varies, of course. We have one physician who’s fond of and may prescribe bioimpedance measurements [...]. But it’s mostly us, the nurses, who want the aid, for dry weight determination. [Nurse, CHU]

We always consult the physician and tell what it [the bioimpedance device] has shown, and then you can see how the patient […], what the other parameters for the assessment of dry weight, you can discuss it. It is not always that we determine dry weight after what the BCM shows, but it may indicate that it should be higher and then the physician may increase it. [Nurse, UHU]

### Organizational context

#### Barriers

One frequently mentioned barrier to use of bioimpedance was lack of structure. There were large variations in routines regarding when to use it, how to interpret results and how to follow up. Some units had guidelines for utilization, but due to high workloads and a shortage of trained staff, bioimpedance measurement was not a priority. Having to wait for the device if someone else was currently using it could interrupt workflow, but this barrier did not have a high impact on use.

To prevent the spread of infection, one patient with a multi-drug resistant infection had been isolated. Equipment brought to the room had been kept to a minimum; consequently, bioimpedance measurement had not been performed preventively, and the patient gradually developed pulmonary edema.

##### Structures

ᅟ

We [the nurses] may have different ways of doing it. [...] We have no guidelines or so on how to do. No follow-up system. [Nurse, UHU]

Since there’s no routine to measure regularly, it’s up to individual nurses [...], those who are a little more alert and interested. [Nephrologist, UHU]

##### Capacity

ᅟ

The problem is when nurses quit and are replaced; there will be new nurses. To do measurements, it takes some commitment and motivation, for it to be of any benefit. [Nephrologist, UHU]

Some people prioritize other things. Going and getting a machine to measure might sometimes be met with resistance. As for now, with 24 extra patients and a shortage of six staff members, you have to set priorities, and it’s not going to be bioimpedance measurement. [Nurse, UHU]

##### Care process

ᅟ

In our unit, sometimes - in the morning when starting the treatments it's quite hectic – the device is occupied, because it’s used every day. So it's always in another room, and sometimes you may not want to wait the extra five minutes, so you start the treatment and you'll do the measurement the next time. That may be a reason why you skip it. [Nurse, CHU

##### Regulations

ᅟ

A patient was kept isolated due to a multi-drug resistant infection. He - without any known new infarction or so - began to develop pulmonary edema. It has been ten to twenty years since I experienced dialysis patients in that situation, but he had not been measured then. [Nephrologist, UHU]

#### Facilitators

Small units had higher capacity for organizational change. For example, participants from a satellite clinic described successful implementation of bioimpedance in clinical practice despite absence of written guidelines.

##### Capacities

ᅟ

Our unit’s pretty small [...]. If there’s a note in the record that says ‘today it’s time for bioimpedance measurement,’ then the person taking care of the patient will perform the bioimpedance measurement. [Nurse, SU]

I'm in a small unit [PD-unit]. It's easier for us to standardize than it is in the blood dialysis unit. [Nurse, CHU]

Considering those [patients] who are very stressed and want their dialysis treatment to get started. If we absolutely want to measure bioimpedance, we usually ask them to come a little earlier, then they can start their dialysis session at the same time as usual, and that usually works. [Nurse, UHU]

## Discussion

The importance of adequate fluid volume management in hemodialysis patients is well established, and a number of technologies are now available to aid assessment of fluid status of which bioimpedance spectroscopy has been most extensively studied [8–20]. However, many dialysis centers lack an agreed fluid management policy, and studies show access to bioimpedance devices in the clinics may not have impact on practice patterns [21, 22]. An evident barrier for clinical use of bioimpedance, which may subjectively prohibit routine measurements, was professionals’ perception of insufficient credibility; several participants of the focus groups found the method unreliable, due to inconsistent measurement results. However, all methods for assessment of hydration status perform best when measured serially and when performed in conjunction with other methods of volume assessment [7, 12–14, 33], but not all study participants were aware of the importance of serial bioimpedance measurements. Users’ questioning the underlying evidence is a known barrier for implementation, and as evidence often focuses on patients with single diseases and excludes complex patients, practical applicability may be limited. [34–36].

In some clinics, use of bioimpedance in daily practice depended on individual initiatives. Bioimpedance had not been introduced strategically, but through passive dissemination of information, which is generally ineffective [24, 25, 37]. Thus, awareness of the potential benefits of bioimpedance [10, 11, 16, 18, 19] was insufficient. Recommendations for use of bioimpedance have changed over time [38], but there were diverse opinions on how to use bioimpedance, e.g. in patients with a pacemaker, amputees, or for assessing nutritional status. These findings indicate the necessity of channels to provide new and updated research recommendations [24, 39, 40].

Lack of collaboration between different types of professionals and deficient congruency in recommendations were perceived as barriers. Some nurses reported limited self-efficacy in using bioimpedance and interpreting the results due to lack of preexisting knowledge, but in units where dieticians contributed knowledge, participants expressed a higher degree of self-efficacy. Inter-professional collaboration may be critical to the provision of efficient health care and has the potential to increase self-efficacy [41].

Some participants perceived patients’ preference to start the hemodialysis session without delay as a barrier for using bioimpedance. However, as other participants denied that this was a barrier, professionals may also have misconceptions about patient values [30]. Software for visualization of bioimpedance results was found helpful in interactions with patients, and motivation and curiosity among well-informed patients were an incentive for use of bioimpedance [42, 43].

Several participants in the focus groups, the physicians in particular, found the device attractive, as it had contributed to increased knowledge about hydration status and put the subject of dry weight on the agenda. Participants had experienced advantages in clinical practice and found patients to be motivated, resulting in the professionals’ own motivation to change their practice. Attractiveness and experience of advantages in practice are characteristics considered crucial for successful implementation of an innovation [26]. Hence, implementation of bioimpedance in clinical practice has theoretically a good chance for success. However, in most units prime initiators of bioimpedance-measurement were nurses, and use of bioimpedance in the clinical setting was still dependent on certain individuals’ personal interest and dedication. Although some units had developed routines for use of bioimpedance, measurement would not be a priority in periods of high workload and shortage of trained staff. This may indicate that many professionals do not consider bioimpedance a facilitator in daily practice, and inter-professional consensus is missing. Contextual factors, such as hospital size, may also influence successful implementation [44], as the use of bioimpedance had been implemented successfully without systematic implementation strategies in small units.

### Limitations

Due to the qualitative approach, we gained insight into perceived barriers and facilitators, although we cannot appraise the frequency of the identified determinants or their impact on the use of bioimpedance. In order to develop an implementation strategy on a national level or in other countries, a quantitative study on the frequency and impact of identified themes and concepts in this study, could contribute to increased transferability. To prevent inaccuracy in the results, we pilot-tested the questioning route, and used the same moderator in all interviews and for improved confirmability [45], study reporting is based on a rich representation of quotations. Also, as the moderator and assisting moderator debriefed immediately after each session, a tendency to intellectualize [27] was identified in the first focus group, thus the questioning route could be adapted in order to make a clearer distinction between participants’ intended behavior and the setting description.

A limitation of the study is that a selection bias might be inherent due to the strategy to recruit volunteer participants who can best supply information i.e. renal care professionals with experience of using bioimpedance, but whom might also be professionals with most favorable opinions. That is, professionals with unusual experiences and other perceptions might have been missed. There is also a risk, that the study participants’ differences in age and years in profession may have biased the result. The choice to conduct interviews via equipment for telemedicine may have affected dependability, as some users were unaccustomed to the setting. On the other hand, use of telemedicine allowed for interviews with nationwide representatives. A multidisciplinary perspective, including a wide variety of professionals and different types of clinics enhanced credibility, but input from physicians and dieticians was limited due to the small number of participants. We therefore cannot fully assure that we reached saturation on all themes [27, 29] or that all potential perceptions from physicians and dieticians was materialized. However, our aim was not to compare and contrast differences between different professionals perception, and in order to enhance feasibility it is an accepted rule of thumb to plan for three or four interviews when using focus groups for data collection, since the analyst looks for patterns and themes across groups [27]. Moreover, the relative proportions of dieticians, nephrologists and nurses in the study sample do reflect the actuality of the study population well.

## Conclusion

Bioimpedance may contribute valuable support to clinical assessment of hydration status in hemodialysis patients. In this qualitative study content analysis of focus groups interviews with renal care professionals was used to identify perceived barriers and facilitators for use of bioimpedance in clinical practice. A multilevel approach to examining barriers and incentives for change was found to be applicable to the ideas and categories that arose from the data, and determinants, either facilitating or preventing use of bioimpedance, were identified on five levels: 1) the innovation itself, 2) the individual professional, 3) the patient, 4) the social context and 5) the organizational context. Barriers for use were found in the areas of insufficient credibility, lack of awareness, insufficient knowledge, limited self-efficacy, lack of structure and contradictory regulations. However, implementation of bioimpedance have the potential to be successful, as several facilitating factors were found; such as attractiveness of the device and users’ experiences of advantages in practice. Moreover, in units with inter-professional collaboration, participants expressed higher levels of knowledge and self-efficacy, which contributed to motivation to change practice.

## Additional file

Additional file 1: Study reporting was based on the Consolidated Criteria for Reporting Qualitative Health Research (COREQ). (DOC 62 kb)

## Abbreviations

CHU: County hospital unit
SU: Satellite unit
UHU: University hospital unit
